# Sensory Integration Therapy for Preschool Children with Autism Spectrum Disorder and Co-Occurring Intellectual Disability: An Exploratory Single-Group Pre–Post Study

**DOI:** 10.3390/children13040569

**Published:** 2026-04-20

**Authors:** Hirotsugu Nakamura, Kiyomi Tateyama, Kazuyo Nakaoka, Toshihiro Kato

**Affiliations:** 1 Doctoral Program, Graduate School of Comprehensive Rehabilitation, Osaka Prefecture University, 2-1-132 Morinomiya, Joto-ku, Osaka-shi 536-8525, Osaka, Japan; 2 Nishinomiya City Child and Family Center Clinic, 2-77 Takahata-cho, Nishinomiya 663-8202, Hyogo, Japan; 3 Graduate School of Rehabilitation Science, Osaka Metropolitan University, 2-1-132 Morinomiya, Joto-ku, Osaka-shi 536-8525, Osaka, Japan; 4 Graduate School of Life Health Science, Kansai Medical University, 18-89 Uyamahigasshi-machi, Hirakata 573-1136, Osaka, Japan

**Keywords:** autism spectrum disorder, intellectual disability, sensory integration therapy

## Abstract

**Highlights:**

**What is the main finding?**

• Over the 8-week intervention period, sensory integration therapy was delivered once weekly, reflecting the standard Japanese clinical practice; improvements were observed in communication, social skills, and activities of daily living among children with autism spectrum disorder and co-occurring intellectual disability. However, they were subject to multiple sources of bias and methodological limitations, resulting in a high degree of uncertainty.

**What is the implication of the main finding?**

• The changes observed during the sensory integration therapy intervention period remain suggestive rather than conclusive. To further explore the potential effects of sensory integration therapy, future studies should address key methodological challenges, including the inclusion of a control group, expansion of participating sites and sample sizes, and introduction of blinding procedures.

**Abstract:**

**Background:** Occupational therapists often provide sensory integration therapy (SIT) as part of interventions for children with autism spectrum disorder (ASD). However, evidence supporting its effectiveness remains limited. Therefore, this study aimed to explore the potential benefits of once-weekly SIT for children with ASD and co-occurring intellectual disability. **Methods:** A non-blinded single-group pre–post study was conducted using SIT once a week for 8 weeks. Participants were children aged 2–6 years who had been diagnosed with ASD, had a developmental index score of ≤70, and were classified as having severe autism according to the Childhood Autism Rating Scale. Outcome measures included the Goal Attainment Scaling (GAS), Vineland Adaptive Behavior Scales, Second Edition (VABS-II), Short Sensory Profile (SSP), and Parenting Stress Index, Short Form (PSI-SF). Data were analyzed using the Wilcoxon signed-rank test to compare pre- and post-intervention results. **Results:** Ten children completed the full intervention protocol. Changes were observed in some domains of the GAS and VABS-II; however, these findings were characterized by substantial uncertainty and considerable variability across participants. In contrast, no apparent changes were observed in the SSP or PSI-SF. **Conclusions:** The findings of this study do not support the effectiveness of sensory integration therapy (SIT) and should not be interpreted as evidence of intervention-related benefit. Rather, the results should be considered as exploratory observations obtained under real-world clinical conditions. Future research employing more rigorous designs, including the use of control groups, larger sample sizes, and blinded assessments, is required.

## 1. Introduction

Autism spectrum disorder (ASD) is a neurodevelopmental condition defined by persistent impairments in reciprocal social communication and interaction, together with restricted and repetitive patterns of behavior, interests, or activities. Sensory processing difficulties—such as hypersensitivity or hyposensitivity to sensory stimuli—are also frequently observed in individuals with ASD [1,2]. In addition, these characteristic features of ASD have been associated with significantly elevated levels of parenting stress among caregivers, compared with parents of children with other disabilities or those of typically developing children [3].

The prevalence of ASD has been reported to be 2.76% among 8-year-old children in the United States [4] and 3.22% among 5-year-old children in Japan [5], highlighting the importance of providing appropriate support for individuals with ASD in Japan.

According to the 2021 White Paper on Occupational Therapy [6], developmental disorders such as ASD account for 58.6% of all pediatric occupational therapy (OT) cases in Japan. As part of interventions for children and individuals with ASD, occupational therapists (OTRs) often provide sensory integration therapy (SIT).

SIT, developed by American occupational therapist A. Jean Ayres, is an OT approach that is used to assess and treat children’s learning, behavioral, emotional, and social development from the perspective of sensory integration in the brain (sensory integration theory). This theory is based on the hypothesis that the brain’s ability to process sensory information influences learning and behavioral performance [7]. In Japan, SIT is frequently used in OT practice for children with ASD.

Regarding the effectiveness of SIT, the American Academy of Pediatrics has reported a critical lack of scientific evidence supporting its efficacy [8]. Novak et al. classified SIT for ASD as “not recommended” [9], and systematic reviews have indicated that high-quality studies such as randomized controlled trials are limited to fewer than three publications [10,11]. In response to these concerns, recent studies have reported improvements in daily functioning and sensory integration abilities based on randomized controlled trials [12], as well as significant deviations from baseline trends in single-case studies [13,14,15]. Sensory-based interventions more broadly have been criticized for lacking standardized methods to assess treatment fidelity, raising concerns regarding reproducibility [16]. However, in these previous studies, the Ayres Sensory Integration Fidelity Measure (ASIFM) [17]—a scale developed to support occupational therapists in delivering sensory integration therapy in accordance with Ayres’ principles—was used, allowing for the evaluation and assurance of intervention fidelity. Furthermore, a systematic review demonstrated effects across multiple domains, including motor function, cognitive function, social skills, and communication, and reported improvements in activities of daily living and quality of life [18]. The primary outcome measures used in these previous studies focused on functional impairments in daily life—such as social functioning, self-care skills, and adaptive behavior—as well as individualized goals specific to each participant. Furthermore, the primary aim of treatment was not direct intervention for functional difficulties or individualized challenges in daily life, but rather the promotion of underlying sensory integration functions. This framework suggests a theoretical link between sensory integration processes and functional impairments in daily life as well as individualized challenges. Sensory integration theory is based on the hypothesis that the processing and integration of sensory input derived from movement and the environment form the foundation for action planning and adaptive interaction with the environment, thereby supporting the development of learning and behavior [7]. Accordingly, intervention is intended to address each child’s individualized functional difficulties. Within this theoretical framework, targeting sensory processing and integration to facilitate adaptive responses in each child is assumed to influence daily functioning and individualized challenges. Therefore, SIT is theoretically expected to be reflected in changes in adaptive behavior and individualized goals; however, the causal relationship between these processes and outcomes remains unclear.

However, in previous studies, the children with ASD who were included were either those without co-occurring intellectual disability (ID), or studies did not specify ID status as a selection criterion. In some cases, intelligence or developmental levels were not assessed using measures such as Intelligence Quotient (IQ) or Developmental Quotient (DQ), or inclusion criteria were restricted to children with IQ above a certain threshold (e.g., ≥70). Furthermore, several studies did not include ID status or IQ/DQ as part of their eligibility criteria [12,18]. As a result, to our knowledge, no prior studies have specifically examined SIT in children with ASD and co-occurring ID as a defined target population. Therefore, research on SIT specifically targeting children with ASD and co-occurring ID remains limited, and the characteristics of this population and their potential responses to intervention have not yet been sufficiently clarified.

ID is a developmental disorder characterized by deficits in both intellectual and adaptive functioning across conceptual, social, and practical domains [1]. The comorbidity rate of ID among children with ASD has been reported as 37.9% in the United States [4] and 36.8% in Japan [5]. Children with ASD and co-occurring ID exhibit greater challenges and impairments across a wide range of behaviors and skills compared with those who have either [19], making early intervention essential. Given these considerations, it is important to examine the potential applicability of SIT for children with ASD and co-occurring ID, as well as the characteristics of change associated with the intervention.

Although SIT has previously been reported to be provided at least twice per week [18], in clinical practice in Japan, it is offered not more than once per week [20]. The frequency of occupational therapy sessions in Japan is restricted by the medical insurance system, child disability support system, systemic reimbursement limitations, and a shortage of specialists [6,21], resulting in sessions typically being provided less than once per week. However, the effectiveness of SIT provided less frequently than once per week, reflecting actual practice conditions in Japan, remains uninvestigated. These constraints are largely attributable to systemic limitations, making it difficult to implement SIT at a frequency of two or more sessions per week, as reported in previous studies.

Accordingly, the present study aimed to explore the potential for change associated with SIT delivered at the frequency commonly used in Japanese clinical practice (once weekly) among children with ASD and co-occurring ID. A single-group pre–post study design was employed. Given the limited evidence regarding SIT delivered at such a low frequency (once weekly), no specific predictions were made regarding the nature or magnitude of change. Therefore, the pre–post assessments in this study were not intended to test predefined effects, but rather to explore what types of changes might be observed under real-world clinical conditions. Based on previous SIT research, the characteristics of children with ASD and co-occurring ID, and the presence of sensory processing difficulties associated with ASD, we hypothesized that changes might be observed in individualized goals, adaptive behavior, and sensory processing. While adaptive behavior can be assessed using standardized developmental measures, we considered that individualized measures would be necessary to capture ASD-specific communication and behavioral characteristics in children with co-occurring ID. In addition, to explore the potential impact of these changes on caregivers, parenting stress was included as an outcome measure.

## 2. Materials and Methods

### 2.1. Study Design

This study was conducted at the Nishinomiya City Child and Family Center Clinic, a core medical and developmental support facility located in Nishinomiya City, Hyogo Prefecture, Japan, which has a population of approximately 480,000. The Center provides medical and rehabilitation services for children under 18 years of age residing in Nishinomiya City. Its multidisciplinary team, comprising physicians, nurses, physical therapists, occupational therapists, speech language hearing therapists, and psychotherapists, provides medical care and developmental rehabilitation for children and families facing physical or mental developmental challenges.

Nishinomiya City has approximately 90 child development support centers that provide services to preschool children with developmental challenges through individualized instruction and group-based activities. In Japan, “ryoiku” refers to a comprehensive system of support for children with developmental disabilities, encompassing developmental, family, and community-based living support [22]. Each center is required to employ licensed preschool educators and instructors and offer several programs aimed at fostering fundamental daily living skills and promoting adaptation to group activities. Service users undergo an assessment process conducted by Nishinomiya City and select a center based on their family circumstances and individual needs.

Ethical approval for this study was obtained from the Committee on Research Ethics, Graduate School of Comprehensive Rehabilitation, Osaka Prefecture University (Approval No. 2021-217) on 30 March 2022. The study was conducted as a preliminary investigation prior to a planned non-randomized controlled trial on the therapeutic effects of SIT (scheduled study period: 30 March 2022, to 31 March 2027). Written informed consent was obtained from the parents of all participating children after providing both oral and written explanations of the study. Participants were informed that they could withdraw from the study at any time without providing a reason. The preliminary study was carried out between May 2023 and May 2024. Following ethical approval, coordination with the study site required additional time to address issues such as ensuring fairness between clients on the facility’s waiting list and those currently receiving services.

Consequently, it was determined that public registration of the clinical trial should take place after these issues had been resolved. Therefore, registration with the University Hospital Medical Information Network (UMIN) was completed after conducting this exploratory preliminary study (UMIN000059427) and was approved by the Committee on Research Ethics of the Graduate School of Comprehensive Rehabilitation, Osaka Prefecture University.

### 2.2. Eligibility Criteria

Participants were selected according to the following inclusion criteria:

(1) Diagnosis of ASD by a pediatrician at the Nishinomiya City Child and Family Center Clinic based on the Diagnostic and Statistical Manual of Mental Disorders, Fifth Edition (DSM-5).
(2) Age between 2 years 0 months and 6 years 9 months of age. The study included only children who would remain preschool-aged throughout the treatment and assessment period.
(3) ASD severity classified as severe (≥37 points) using the Childhood Autism Rating Scale, Second Edition–Standard Version (CARS2-ST).
(4) Developmental Quotient (DQ) of ≤70. Because it is often difficult to administer intelligence tests to children with ASD and co-occurring ID, DQ was used as an index of intellectual ability.

Exclusion criteria were as follows: (1) presence of an evident physical disability such as cerebral palsy; (2) medical conditions such as epilepsy or heart disease that contraindicate physical activity; (3) current or past receipt of SIT provided by an occupational therapist; and (4) children with significant challenges in family background, such as suspected abuse, inappropriate caregiver–child interactions, or parental conditions that substantially interfere with daily functioning. For criterion (4), given the potential difficulty in making such judgments, eligibility was determined through consultation among the clinic director, the attending physician, the lead occupational therapist, and other relevant therapists, in order to ensure that participation in the study would not interfere with the child’s access to clinical care and developmental services.

### 2.3. Procedures

**Figure 1:**
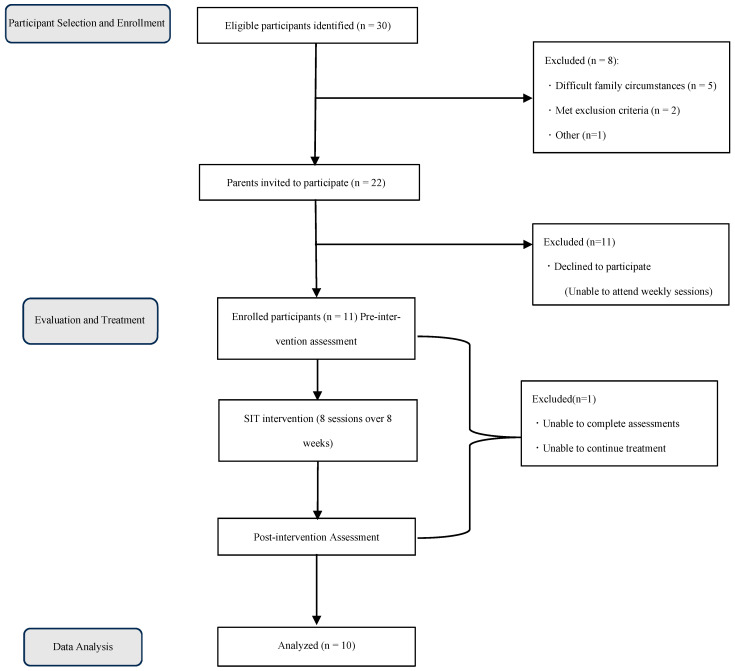
Study participant flow chart. The gray-outlined boxes indicate the study phases, while the black-outlined boxes indicate participant flow steps.

Figure 1 presents the study procedure and study population size. Regarding the selection procedure, information was first collected from medical records and relevant staff members, including pediatricians, speech–language therapists (STs), and nursery staff, for children scheduled to undergo an initial OT evaluation. Based on this information, inclusion criteria (1), (2), and (4) were confirmed, CARS2-ST scoring was conducted for criterion (3), and exclusion criteria were reviewed. Subsequently, for children who met the preliminary criteria, observations were conducted during the initial OT session to reconfirm CARS2 scoring. Following this process, children who met all eligibility criteria were recruited through their parents. For those whose guardians consented to participate, the responsible OTR conducted pre-treatment assessments and measurements in consultation with a pediatrician and an ST. These assessments incorporated information obtained during participant selection, as well as additional data collected through parental interviews and questionnaires.

Previous studies have suggested that at least six weeks of intervention may be required to observe changes associated with SIT in children with ASD [12,23]. In contrast, children with ASD and co-occurring ID are reported to exhibit greater challenges across various behaviors and skills compared with those with ASD or ID alone [14].

Moreover, in children with neurodevelopmental conditions, differences in adaptive behavior may widen over longer developmental periods [24]. Considering these developmental characteristics, it is unlikely that substantial changes attributable to natural development would occur within a relatively short observation period of several weeks.

Based on these considerations, the intervention period was set at 8 weeks to allow observation of potential changes while minimizing the influence of natural developmental progression. In addition, to ensure fairness among service users at the study site, each session was limited to 40 min.

Following intervention completion, post-treatment assessments and measurements were conducted through parental interviews and questionnaires.

In addition, the same occupational therapist was responsible for both delivering the intervention and conducting the assessments, and no blinding procedures were implemented. Therefore, the results may have been influenced by observer bias, expectancy bias, and related sources of measurement bias.

### 2.4. Sensory Integration Therapy

SIT was conducted by a certified and registered occupational therapist (No. 141) with 17 years of clinical experience from the Japanese Society for Sensory Integration. This certification is awarded to professionals in Japan who complete specialised training in the assessment and intervention methods of SIT and are accredited by the society [25]. The intervention was conducted in a sensory integration room within the clinic used by OTRs, which was equipped with essential apparatus for SIT, including a trampoline, swing, and ball pool.

The ASIFM was referenced in planning the intervention. However, because an independent fidelity assessment system has not yet been established in Japan, formal fidelity evaluation was not conducted. Therefore, the consistency and reproducibility of the intervention in this study may be limited, and the findings should be interpreted with caution. Treatment goals and intervention strategies were developed with reference to the ASIFM and were discussed collaboratively with two co-investigators and the attending pediatrician at the clinic. The two co-investigators are OTRs with more than 10 years of clinical experience, including consultation for children with ASD and co-occurring ID.

Given the characteristics of the study participants, namely children with ASD and co-occurring ID who exhibited various behavioral difficulties [19] and were classified as having severe ASD according to the CARS2, the intervention emphasized one of the core principles of SIT: establishing a therapeutic relationship with the child. To achieve this, the initial phase of intervention emphasized engagement based on the child’s physiological needs and intrinsic motivation. Opportunities for sensory experiences were provided to facilitate shared attention and affective engagement through activities. When the child’s intentions or preferences were unclear, priority was given to ensuring a sense of safety and stabilizing sensory input from the environment. For example, for children with high anxiety in novel situations, strategies included allowing observation from a caregiver’s proximity, prioritizing equipment aligned with the child’s visual focus, and structuring activities to be easily understandable and achievable. During activities, task demands and sensory input were continuously adjusted according to the child’s pace to maintain interest and a sense of accomplishment. Direct interaction included verbal labeling of the child’s focus of attention and actions, supporting awareness of the environment and internal states. Positive responses such as eye contact, vocalizations, or facial expressions were reinforced by recreating similar interactional contexts.

Individual differences among participants were addressed by focusing on the quality of intrinsic motivation and its changes over time. The quality of intrinsic motivation was conceptualized with reference to key elements of sensory integration therapy, including sensory experiences, organization of behavior, and interaction with others, as reflected in the ASIFM framework. Activities were designed to incorporate elements that each child was naturally inclined to engage with, while providing support to facilitate a sense of achievement.

For example, when a child showed a preference for swinging, variations in the quality and intensity of vestibular input were introduced to allow initial passive enjoyment, followed by opportunities for the child to actively control the movement and make decisions, including requesting support from the therapist. As engagement progressed, attention was given to changes in the child’s intrinsic motivation, and activities were adjusted accordingly. For instance, when the child shifted from passively enjoying movement to actively controlling it, more challenging tasks were introduced, such as controlling movement while interacting with objects.

As the intervention progressed, the child’s responses during sessions were reviewed, and both the treatment content and evaluations were adjusted as needed based on written records and video reviews.

### 2.5. Tools Used for Participant Selection

For participant selection, the CARS2-ST was administered, and KSPD results were reviewed.

The CARS2-ST is a revised version of the original CARS, developed to differentiate children with ASD from those with other developmental disorders. It is a scale used to diagnose ASD, differentiate it from other disorders, and further classify the diagnosed disease as mild, moderate, mild-to-moderate or severe.

The items incorporate the core features of autism described by Kanner, additional characteristics identified by Creak, and supplementary scales useful for evaluating symptoms in young children. The measure has also been validated as capturing the core symptoms described in previous editions of the DSM-5. To calculate the total score, scores below 30, 30–36.5, and 37–40 are classified as non-ASD, mild-to-moderate ASD, and severe ASD, respectively [26].

Ratings are based on observations made in various contexts, such as during psychological testing or at the child’s regular institution, along with reports from parents, comprehensive clinical records, or an integration of these sources.

In this study, assessments were conducted using clinical records, behavioral observations, and interviews with staff members at the Nishinomiya City Child and Family Center Clinic.

The KSPD is a standardized developmental assessment widely used in Japan [27,28]. It is most commonly employed during health checkups for infants and young children whose developmental stage makes it difficult to administer Wechsler-type intelligence tests [29], and it is used in determining eligibility for the Ryoiku Techo (rehabilitation certificate) issued to children with ID.

This assessment evaluates three developmental domains. The Postural-Motor (P-M) domain evaluates motor function, the Cognitive-Adaptive (C-A) domain assesses cognitive abilities including nonverbal reasoning and visuospatial perception, while the Language-Social (L-S) domain examines communication encompassing interpersonal relationships, social skills, and language abilities. A developmental quotient (DQ) is calculated for each of these three domains and for an overall composite score [30]. The DQ derived from the KSPD is considered a useful indicator for evaluating a child’s developmental level [31].

In this study, the results of the KSPD previously administered at the Nishinomiya City Child and Family Center Clinic or at child guidance centers were reviewed.

### 2.6. Outcome Measures and Assessment Tools

Because OT emphasizes the individuality of each child, “individualized treatment goals” were included as a measurement item. As children with ASD and ID often exhibit a wide range of behavioral challenges and difficulties [18], “behavior” was also selected. Furthermore, since children with ASD frequently experience sensory issues such as hypersensitivity or hyposensitivity in addition to their core characteristics [1,2], “sensation” was included. Finally, as parents of children with ASD have been reported to experience significantly higher levels of child-rearing stress compared with parents of children with other disabilities or typically developing children [3], “child-rearing stress” was also assessed. Thus, four domains were measured: individualized treatment goals, behavior, sensation, and child-rearing stress.

The corresponding assessment and measurement tools used for each are described below.

### 2.7. Outcome Measures

#### 2.7.1. Evaluation of Therapeutic Goal Attainment: Goal Attainment Scaling & Canadian Occupational Performance Measure

Goal Attainment Scaling (GAS) is a standardized evaluation method in which individualized rating scales are created for each client to measure the degree of goal achievement within their treatment program [32]. It has demonstrated high reliability and responsiveness across a variety of contexts, including pediatrics and cognitive rehabilitation [33,34,35,36,37,38,39], and is also recommended for evaluating the effectiveness of OT and SIT interventions [40].

In GAS, three to five treatment goals that are meaningful to the client are established based on information gathered from the client, family members, and professionals involved in the client’s care, such as those in medical, health, educational, or childcare settings. Each goal is rated on a five-point scale ranging from −2 to +2, where −1 represents the current performance level and 0 represents the expected level of achievement.

There are several methods for scoring GAS, but we used the method described by Gordon et al. [34] in this study and calculated the modified GAS score. A T-score of 50 indicates that the expected outcome was achieved, scores above 50 indicate outcomes exceeding expectations, and scores below 50 indicate outcomes below expectations.

In this study, “goals meaningful to the client” were identified using the Canadian Occupational Performance Measure (COPM). The content and gradation of each goal were determined collaboratively with two co-researchers who were OTRs with 10 or more years of clinical experience with ASD and co-occurring ID, as well as experience conducting outcome studies using GAS.

The COPM was developed to ensure the quality of occupational therapy by identifying activities that are meaningful to the client and tracking the client’s perception of performance in those activities over time. It is an individualized, client-centered outcome measure that has demonstrated high validity, reliability, usefulness, and responsiveness [41,42].

In this study, the COPM was used to identify activities meaningful to each client, determine their perceived importance, and incorporate this information into GAS.

#### 2.7.2. Behavioral Assessment: Vineland Adaptive Behavior Scales, Second Edition (VABS-II)

The VABS-II is an assessment tool used to evaluate adaptive behavior and has demonstrated high validity and reliability. The Adaptive Behavior Assessment is divided into four domains—communication, daily living skills, socialization, and motor skills—each comprising two to three subdomains.

Composite and domain scores for adaptive behavior are calculated as standard scores. Subdomain scores are expressed as v-scale scores (mean = 15, standard deviation [SD] = 3), which allow for detailed identification of low-level performance that significantly limit adaptive functioning in individuals with disabilities such as ASD or ID [43].

The evaluation is conducted through semi-structured interviews with the parents of the children. To improve interview efficiency, a Parent/Caregiver Questionnaire is provided beforehand to gather preliminary information [44].

In this study, the Adaptive Behavior Assessment was administered to the parents of participating children. Because children with ASD and co-occurring ID often exhibit uneven developmental skills [45,46] and ASD-specific behavioral patterns may not align well with standardized items, the semi-structured interviews required considerable time. Given these factors, securing sufficient time for interviews was expected to be difficult within the practical constraints of the research setting. Moreover, the Japanese version of the VABS-II does not include the Parent/Caregiver Questionnaire. Therefore, in this study, information was collected through semi-structured interviews, supplemented by individualized questionnaires developed for each participant. The questionnaire items were organized according to the subdomains of the four VABS-II domains, and responses were recorded in free-text format. As the psychometric properties (e.g., reliability and validity) of these additional items have not been established, the data were not treated as standardized measures but were used descriptively to capture individual changes.

#### 2.7.3. Sensory Assessment: Short Sensory Profile

The SSP is a shortened form of the Sensory Profile (SP), developed to assess sensory processing abilities and their effect on daily functioning. This questionnaire enables rapid assessment in both clinical practice and research [47].

Both the original and Japanese versions have been confirmed to have sufficient reliability and validity [48].

The SSP consists of nine section scores and an overall total score that is particularly important for clearly identifying a child’s sensory processing abilities. When the total score falls within the “much higher than others” range (mean +2 SD or above), it may indicate difficulty adapting to the environment and a potential for self-directed or outwardly disruptive behaviors. When the score falls within the “higher than others” range (mean +1 SD to +2 SD), it may suggest that sensory processing difficulties are interfering with daily life.

#### 2.7.4. Assessment of Child-Rearing Stress: Parenting Stress Index, Short Form

The Parenting Stress Index Short Form (PSI-SF) is a shortened version of the Parenting Stress Index (PSI) [49], a screening tool designed to identify parents who may be at risk of difficulties in child-rearing or who have children with developmental risks.

The Japanese version of the PSI-SF consists of 19 items with a two-factor structure: “Child Domain” and “Parent Domain”. The Child Domain comprises nine items, such as “My child is so active that it tires me” and “My child has less concentration than other children”. The Parent Domain consists of ten items, such as “I enjoy being a parent” and “I often feel that I cannot handle things well”. The Japanese version has been shown to have adequate reliability and validity [50] (pp. 85–92).

### 2.8. Sample Size

This exploratory study aimed to gather preliminary data to inform a future definitive trial. Therefore, a formal sample size calculation based on hypothesis testing for treatment effects was not considered mandatory. In exploratory research, sample size is appropriately determined by study objectives, practical constraints, and the information needed to inform subsequent research planning [51,52]. Furthermore, methodological guidance on pilot studies suggests that including approximately 10–30 participants is generally appropriate for obtaining preliminary estimates of feasibility and variability to inform future trials [51,53]. Therefore, in line with the exploratory nature of this study, the sample size was determined to allow observation of individual changes during the intervention period and to characterize variability in the outcome measures. Feasibility was also considered, and a sample size of 10 participants was selected.

### 2.9. Statistical Analysis

As this study was exploratory in nature and the sample size was limited, statistical analyses were conducted primarily for descriptive purposes and to identify general trends in the data.

The following assessment tools were used to examine changes over the measurement period. Therapeutic goal attainment was evaluated using GAS, behavioral changes using the VABS-II, sensory processing abilities using the SSP, and changes in child-rearing stress using the PSI-SF.

The Shapiro–Wilk test was first performed to assess normality. As some variables, including GAS and VABS-II, were not normally distributed, pre- and post-intervention differences were analyzed using the Wilcoxon signed-rank test. The level of significance was set at *p* < 0.05 (two-tailed). In accordance with the properties of the Wilcoxon test, cases with a difference of zero were automatically excluded from the analysis. In the present study, the effective sample size for the Wilcoxon signed-rank test ranged from 7 to 10. However, because the primary objective was to capture overall trends in change across all participants, the effect size (r) was calculated using the total sample size (n = 10), and confidence intervals were calculated accordingly based on the same total sample size. All statistical analyses were conducted using IBM SPSS Statistics, version 28.0.1.0 (142).

Furthermore, the statistical analyses in this study were not intended to examine causal relationships, but rather to explore patterns of observed change. In addition, given the inability to adequately account for individual differences and potential confounding factors, including concurrent therapeutic services, the findings should be interpreted with caution.

## 3. Results

### 3.1. Participants

From the screening process, 30 children met the eligibility criteria. Based on information obtained in advance from staff members and medical records, eight children were excluded for the following reasons: difficult family circumstances such as abuse or parental illness (n = 5), meeting exclusion criteria (n = 2), or other reasons (n = 1). Participation was subsequently offered to 22 children, of whom 11 consented while the remaining 11 declined owing to difficulty attending weekly sessions. Of the 11 participants who received SIT, one was unable to complete both pre- and post-intervention assessments. The remaining 10 participants completed all eight sessions of SIT (once weekly) as well as the pre- and post-intervention assessments. Among these, one participant consistently arrived approximately five minutes late for each session, resulting in slightly reduced intervention time (Supplementary Table S2, Case 10).

The mean CARS2 score was 39.6 ± 1.7, with all participants classified as having severe ASD. Based on pre-intervention VABS-II results, the mean composite adaptive behavior score was 52.9 ± 9.8, corresponding to below −2 SD. Among the four domains, the lowest scores were observed in socialization (Table 1).

Detailed information on participants’ age, DQ, and CARS scores is presented in Supplementary Table S1. Details of pre–post changes for each assessment tool are provided in Supplementary Table S2.

No changes were planned in the children’s affiliations or individual ryoiku programs during the intervention period.

### 3.2. Outcome Measure

#### 3.2.1. Evaluation of Goal Attainment: GAS

The modified GAS score was calculated based on the attainment of three to four individualized goals established for each participant.

Table 2 presents a summary of the GAS and COPM goals formulated based on the primary concerns of the parents, while Table 3 provides specific examples for each goal category. Table 4 illustrates an example of GAS implementation and scoring.

Post-intervention results showed a mean score of 61.2, compared with the benchmark score of 50, which represents the expected level of goal attainment. A pre–post difference was observed (*p* = 0.005), with an effect size of r = 0.889 (95% CI: 0.269–1.00) (Table 5).

#### 3.2.2. Behavioral Assessment: VABS-II

A difference in the Adaptive Behavior Composite score was observed between pre- and post-intervention assessments (*p* = 0.005), with an effect size of r = 0.893 (95% CI: 0.274–1.00). Similarly, increases in scores were observed in the domains of communication (*p* = 0.012), daily living skills (*p* = 0.017), and socialization (*p* = 0.005). The confidence intervals for the effect sizes ranged from small to large. In contrast, although a difference was observed in the reading and writing subdomain (*p* = 0.035), the score decreased (Table 5).

No significant differences were observed in the motor skills domain or its subdomains. Furthermore, within domains that showed overall differences, several subdomains—particularly within daily living skills and socialization—did not demonstrate significant changes.

#### 3.2.3. Sensory Assessment: SSP

No difference was observed in the total score between pre- and post-treatment. Regarding changes in score classification, one participant shifted from “Very High” to “High”, whereas three participants shifted from “High” to “Very High”, and one participant shifted from “Average” to “High”. Among the five participants whose classification did not change, all were categorized as “High”.

#### 3.2.4. Parental Stress Assessment: PSI-SF

No difference was observed in the total score between pre- and post-intervention assessments. The mean percentile rank was approximately 87 before the intervention and 88 after, indicating little to no change (Table 5).

#### 3.2.5. Individual Variability in Change (Supplementary Table S2)

Changes varied across individuals for all outcome measures. The magnitude of change in GAS T-scores showed a wide range (+15 to +41), indicating substantial individual variability. In contrast, changes in VABS-II composite scores were relatively small and also varied across participants (+1 to +6). SSP total scores showed both increases and decreases, with no consistent pattern of change in sensory processing. Similarly, PSI-SF scores demonstrated both increases and decreases, with no consistent pattern observed.

## 4. Discussion

### 4.1. Characteristics of the Participants

Among the 10 participants who completed the pre–post comparison, nine were classified as having mild intellectual disability (DQ 51–70) and one as having moderate intellectual disability (DQ 36–50), while no participants were classified as having severe (DQ 21–35) or profound (DQ ≤ 20) intellectual disability. In the general population of individuals with intellectual disability, approximately 85% are classified as mild, 10% as moderate, and 5% as severe or profound [54,55]. Thus, the predominance of mild and moderate cases in the present study is consistent with these general distributions. However, no participants with severe or profound intellectual disability were included, and the sample was therefore skewed toward milder levels of intellectual disability.

### 4.2. Considerations Regarding the Attainment of Individual Treatment Goals

Among the major goal categories (Table 1), “communication” was the most frequent, accounting for approximately 54% of all GAS goals. These goals were primarily related to the features of ASD: deficits in social communication, and restricted and repetitive patterns of behavior, interests, or activities, which influence relationships with others, and participation in group settings. Thus, they were likely to reflect parents’ primary concerns and become central treatment goals.

The second most frequent category, “activities of daily living (ADL)”, comprised approximately 19% of all GAS goals. The content of these goals was related to “impairments in adaptive functioning in practical domains”, a core feature of ID, and was largely reflected in delays or non-acquisition of activities of daily living. As such, these areas were relatively likely to be identified as primary caregiver concerns and treatment goals.

Regarding changes in the modified GAS scores, a positive pre–post difference was observed. However, the confidence intervals for the effect sizes ranged from small to large, indicating substantial uncertainty. In addition, many outcomes in the VABS-II, SSP, and PSI-SF did not show significant differences. Taken together, the observed change in modified GAS scores may have been influenced by the non-blinded study design and the use of interview-based assessments, increasing the risk of detection bias, expectancy bias, and respondent bias. As a result, the magnitude of change may have been overestimated. Furthermore, as GAS is an individualized outcome measure, its results may have limited generalizability compared with standardized developmental measures. Therefore, the findings should be interpreted in conjunction with results from other assessment tools.

### 4.3. Considerations Regarding Behaviour

Among the domains with observed changes, the communication and socialization domains shared conceptual overlap with the individualized treatment goals (Table 3), particularly in terms of receptive and expressive language abilities and relationships with others. Similarly, the daily living skills domain corresponded to the individualized goal category of ADL (Table 3), as both focused on the acquisition and performance of activities of daily living. In these areas, the findings suggest the possibility of change. However, the results may have been influenced by detection and performance bias owing to the lack of blinding, as well as by the use of supplementary individualized questionnaires that may have affected scoring. Given the substantial uncertainty reflected in the wide confidence intervals, it is also possible that the magnitude of change was overestimated. Furthermore, both the GAS and VABS-II were administered using similar methods, individualized questionnaires and semi-structured interviews, which may have increased the risk of common method variance. Accordingly, the observed consistency and associations between results may, in part, reflect shared measurement methods rather than true convergence across constructs.

The observed changes were primarily in functions that typically emerge in early developmental stages. This may be explained by the relatively young age of participants (mean 4.7 ± 0.7 years) and their developmental level (DQ 64.3 ± 4.5), suggesting that developmental immaturity may have contributed to increased responsiveness to intervention or susceptibility to natural developmental change. In addition, given the severity of ASD and variability in condition, this population may be prone to fluctuations in performance, increasing variability in measurement. These characteristics may also increase the likelihood of regression to the mean, suggesting that some of the observed changes may reflect statistical rather than true effects. However, this study did not include detailed analyses of maturation effects or regression to the mean, and the potential influence of these factors should be interpreted with caution.

In contrast, a decline was observed in the reading and writing subdomain. As reading and writing skills generally begin to develop after approximately 3 years of age [56], the results may suggest that, in higher-order communication functions, the developmental gap relative to typically developing peers may have widened.

Many subdomains did not show observable changes, including all three subdomains of daily living skills, two of the three socialization subdomains, and both motor skill subdomains. Despite observed differences at the composite and domain levels, the lack of consistent changes at the subdomain level suggests substantial individual variability. This variability may reflect differences in the types of support and services selected by caregivers based on each child’s needs, potentially contributing to heterogeneous outcomes at the subdomain level while producing broader trends at higher levels of aggregation.

### 4.4. Considerations Regarding Sensory Function

At the pre-intervention assessment, seven participants were classified as “High” or “Very High”, indicating clinically significant sensory processing difficulties. However, no statistically significant changes were observed following the intervention. In contrast, changes in mean scores and score classifications indicated a tendency toward worsening sensory processing difficulties after treatment.

Overall, the findings of this study did not suggest an improvement in sensory processing difficulties associated with SIT. Regarding the observed trend toward worsening, sensory characteristics in children with ASD are generally considered to be relatively stable and unlikely to deteriorate markedly over time [57,58]. Therefore, the apparent decline may reflect individual factors related to the participants or respondents rather than a true treatment-related effect.

Therefore, although the SSP was useful for identifying the severity of sensory processing difficulties, it may not have been suitable for capturing changes over repeated measurements. In addition, it is possible that the 8-week intervention period under the conditions of this study (including SIT and concurrent services) was insufficient to produce measurable changes in sensory processing characteristics.

### 4.5. Considerations Regarding Parental Stress

High levels of parental stress were observed both before and after the intervention, with no significant changes detected over time.

This study used the PSI-SF to explore a correlation between improvement in children’s behavior after SIT with reduced parental parenting stress. However, the present findings did not suggest such a spillover effect.

Therefore, although changes were observed in some domains in the participating children, no corresponding changes were observed in parental stress. Parenting stress is known to be influenced by multiple factors, including family environment and parental psychological characteristics [50] (pp. 15–17), and the magnitude and duration of changes observed in this study may have been insufficient to affect these factors.

Furthermore, although the PSI-SF was useful for assessing parental stress at a given point in time, it may not have been well suited for capturing changes across repeated measurements.

### 4.6. Considerations Regarding Outcome Measures and Sensory Integration

In the communication domain, changes were observed across all VABS-II subdomains, suggesting relatively consistent changes among participants.

Given that communication-related difficulties were frequently identified as individualized treatment goals, these findings may be consistent with previous systematic reviews indicating that individualized measures and communication outcomes are more likely to demonstrate change [11,18].

However, given the substantial uncertainty in the present findings, these results should be interpreted with caution. From a hypothesis-generating perspective, one possible interpretation within the framework of sensory integration theory is that these changes may be related to praxis. Praxis, the ability to plan and execute novel movements and actions, is essential for interacting with the physical environment [7] (pp. 73–87). Ayres also described the relationship between praxis and language, suggesting that both are involved in interaction with the environment, concept formation, and motor planning [59].

Difficulties in adaptive functioning, particularly in activities of daily living, may be theoretically associated with impairments in praxis. Treatment goals identified for participants, such as eating (Table 3) and table-based activities (Table 5), may reflect difficulties in planning motor actions involving physical tools such as utensils, crayons, and scissors—that is, challenges in interacting effectively with the physical environment.

On the other hand, previous studies have reported that the effects of SIT vary considerably depending on the outcome measures used and are often small in magnitude [60]. Similarly, the present study showed numerous non-significant findings, substantial variability across subdomains, and a high degree of uncertainty, indicating several methodological and interpretative challenges. In addition, praxis, as discussed as a theoretical construct, was not directly measured in this study, and this interpretation is not supported by the present data.

### 4.7. Limitations and Future Directions

This study employed a non-blinded, single-group pre–post design and was conducted at a single facility, resulting in multiple potential sources of bias, practical constraints, and limited generalizability.

Regarding participant selection, families with “challenging home backgrounds”, such as those involving abuse or parental illness, were excluded. As a result, the sample may have been biased toward children with relatively higher functioning or more stable circumstances. In terms of the distribution of ID severity, although the overall pattern was generally consistent with commonly reported proportions, no children with severe or profound ID were included, and the sample was skewed toward mild ID. Furthermore, given that the study was conducted at a single site and that considerable individual variability was observed, generalization of the findings to the broader population of children with ASD and co-occurring ID may be limited.

With respect to social services utilized by participants, all children received standard developmental support services in addition to weekly SIT. However, the content, staffing, duration, and number of facilities involved in usual care varied considerably. Systematic documentation, quantification, and analytical control of these factors were not feasible. Although the observed changes may have been influenced by the concurrent provision of usual care, the absence of a control group precluded verification of this possibility. Therefore, the findings of this study should not be interpreted as evidence of the effects of SIT alone, but rather as changes observed under conditions in which multiple forms of support were concurrently provided.

In the statistical analyses, effect sizes and their confidence intervals were calculated using the total sample size (n = 10) to capture overall trends. However, the effective sample size was small (n = 7–10), resulting in wide confidence intervals and substantial uncertainty.

For the GAS and VABS-II, supplementary individualized questionnaires were developed to better capture participant-specific changes. However, the psychometric properties of the additional items included in these questionnaires have not been established. Therefore, findings derived from these data should be interpreted descriptively, and they cannot be used to substantiate change based on standardized measurement alone. Moreover, because both measures relied on parent report and semi-structured interviews, common method variance may have increased, and caution is warranted when interpreting the observed convergence between GAS and VABS-II findings. In addition, the observed changes may have been influenced by natural developmental progression, as well as measurement bias and expectancy bias associated with the non-blinded design.

Given these limitations, the findings of this study should be interpreted as exploratory and indicative of potential changes only. It is not possible to attribute the observed changes to the SIT intervention.

Future research should include controlled study designs with comparison groups receiving usual care without SIT, larger sample sizes, and multi-site recruitment. To ensure intervention fidelity, independent evaluation using the ASIFM by third-party assessors is recommended.

In addition, implementation of blinded outcome assessment is necessary to reduce bias. Regarding outcome measures, GAS and VABS-II may continue to be useful; however, VABS-II should be administered in its standardized semi-structured format. In addition, it is important to consider the use of measures assessing the child’s physical and psychological functions, rather than relying solely on parent-report measures. SSP and PSI-SF may be more appropriately used as baseline descriptive measures of sensory characteristics and parental status, respectively.

## 5. Conclusions

This study aimed to explore the potential for change associated with sensory integration therapy (SIT) delivered once weekly, reflecting typical clinical practice in Japan, in children with autism spectrum disorder (ASD) and co-occurring intellectual disability (ID), using a single-group pre–post design.

Changes were observed in individualized goals and in some domains of adaptive behavior; however, these findings were characterized by substantial uncertainty. No changes were observed in many other outcomes, and considerable variability was noted across participants. Furthermore, the results may have been influenced by multiple limitations, including the concurrent provision of other services, various sources of bias, and constraints related to study implementation.

Therefore, the findings of this study do not support the effectiveness of SIT and should not be interpreted as evidence of intervention-related benefit. The observed changes should be regarded as exploratory observations obtained under real-world clinical conditions. Future studies employing more rigorous designs—such as the inclusion of control groups, larger sample sizes, multi-site recruitment, and blinded assessments—are required to address these methodological limitations.

## Figures and Tables

**Table 1:** Participant Characteristics and Use of Social Resources (n = 10).

Variable	Sub Variable	Frequency
Age (years)	Mean	4.7 ± 0.7
Sex	Male	9
	Female	1
DQ	Mean	64.3 ± 4.5
CARS Score	Mean	39.6 ± 1.7
VABS-II		
Adaptive Behavior Composite Score	Mean	52.9 ± 9.8
Communication	Mean	55.6 ± 13.8
Daily Living Skills	Mean	49.5 ± 13.7
Socialization	Mean	40.6 ± 11.4
Motor Skills	Mean	61.0 ± 16.5
Affiliation		
Nursery School/Kindergarten (n = 2)	Mean Number of Days of Use Number of Children Receiving Additional Support	5/w 2
Child Day Service (n = 3)	Mean Number of Days of Use	3.5/w
Dual Attendance (n = 5)	Mean Number of Days of Use Number of Children Receiving Additional Support	4.2/w + 1.8/w 5
Individual Ryoiku	Number of Users	5
	Frequency of Use	2/w 0.8 h/times

CARS: Childhood Autism Rating Scale; DQ: Developmental Quotient.

**Table 2:** Classification of Activities in the COPM and GAS (n = 10).

Major Goal Category	Subcategory	COPM (n)	GAS (n)
Communication	Interpersonal Relationships	5	7
	Group Participation	6	7
	Expression of Intent	2	3
	Means of Expression	4	2
	Reciprocal Conversation	1	1
ADL	Eating	3	5
	Toileting	2	2
Behavior	Fixation	1	0
	Emotional Regulation	2	1
	Behavioral Flexibility	2	2
	Sustained Attention	3	1
	Situational Awareness	2	1
Motor	Gross Motor Skills	1	2
	Fine Motor Skills	0	1
Sensory	Sensory Hypersensitivity	1	2

COPM: Canadian Occupational Performance Measure; GAS: Goal Attainment Scaling.

**Table 3:** Sample Goals: Participant Challenges and Expected Goals (±0 Level).

Goal Category	Challenge	Expected Goal (±0 Level)
Interpersonal Relationships	During free play in a small-group ryoiku session, the child engages in solitary play and does not attempt to interact with other children.	The child occasionally initiates approach toward other children.
Group Participation	The child has difficulty participating in group activities at kindergarten and tends to withdraw or move away even when encouraged by an assigned support teacher.	With encouragement from the assigned support teacher, the child is occasionally able to remain within the group without avoiding participation (approximately once or twice per week).
Behavioral Flexibility	The child consistently refuses transitions between locations or activities by saying “No.” However, when the next location or activity is presented visually and explained repeatedly, the child is able to transition successfully.	The child still requires visual cues or verbal explanation but is able to transition without repeated prompting.
Eating/Feeding	When eating with a spoon, the child frequently (more than half the time) touches the food with the non-dominant hand to assist in scooping.	The child uses the assisting hand to touch food about half as frequently as before.
Motor Function	When climbing steep stairs, the child holds onto the handrail or touches the step-in front with their hands while ascending.	The frequency of touching the step-in front while climbing stairs decreases by approximately one-third.

**Table 4:** Example of GAS Goal Setting and Scoring.

Goals (n): 4 GAS Score: +3 GAS T-Score: 61	
Goal (1) Attending Kindergarten	
Treatment Plan: To help the child better anticipate situations and improve behavioral flexibility.	
Pre-Intervention Status: The child shows reluctance to attend kindergarten and often cries upon arrival. The child resists entering the classroom and instead runs around outside.	
GAS Goal Setting:	
−2	Attends kindergarten 3 days a week; however, resists attending on all 3 days and occasionally refuses to go.
−1	Attends kindergarten 3 days a week; however, despite resisting attendance on all 3 days, the child does not stay home as a result of this reluctance.
0	Attends kindergarten without resistance on one of 6 days (approximately once every 2 weeks) and does not stay home due to refusal.
+1	Attends kindergarten without resistance on one of 3 days (once per week) and does not stay home due to refusal.
+2	Attends kindergarten without resistance on two of 3 days per week and does not stay home due to refusal.
Post-Intervention Result: −1	
Goal (2) Engaging in Play with Peers	
Treatment Plan: To help the child better anticipate situations and improve body schema awareness.	
Pre-Intervention Status: The child does not actively avoid other children but prefers solitary play and has difficulty participating in group play activities such as chase games.	
GAS Goal Setting:	
−2	Even with adult assistance, the child has difficulty playing in the same space as other children.
−1	With adult assistance, the child can play in the same space as others but has difficulty joining group play.
0	With adult assistance, the child can occasionally participate in group play for a short duration (approximately 2–3 min).
+1	With adult assistance, the child can sometimes participate in the same activity for more than 5 min.
+2	The child is occasionally able to independently join and participate in the same activity without adult assistance.
Post-Intervention Result: +2	
Goal (3) Tabletop Activities	
Treatment Plan: To enhance the child’s understanding and imagery of tool use.	
Pre-Intervention Status: The child is unable to complete even a single step of tabletop activities such as coloring, drawing, or using scissors.	
GAS Goal Setting:	
−2	The child rarely engages in tabletop activities, even with adult assistance.
−1	Even with assistance (repeated verbal prompts, explanations, or demonstration), the child has difficulty completing a single step. For example, coloring within one section or making one cut.
0	With assistance (repeated verbal prompts, explanations, or demonstration), the child is able to complete a single step. For example, coloring within one section or making one cut.
+1	The amount of assistance required to complete one step decreases; the child can engage after one or two verbal prompts, explanations, or demonstrations.
+2	With assistance (repeated verbal prompts, explanations, or demonstration), the child is able to complete two steps. For example, coloring within two sections or making two cuts.
Post-intervention result: +2	
Goal (4) Sensory hypersensitivity and attention/concentration	
Treatment plan: To reduce auditory hypersensitivity and improve sustained attention and concentration.	
Pre-treatment status: During ryoiku activities, the child was distracted by environmental sounds (such as the ventilation fan or voices from outside), often leaving their seat or the room to check the source of the noise.	
GAS goal setting:	
−2	Almost every time a sound occurs, the child stops participating in the developmental activity.
−1	Out of several occurrences of sounds, the child stops participating once to leave their seat and check the source of the sound. After confirming it, they are able to return to the room or seat when individually prompted
0	When distracted by a sound and leaving their seat to check it, the child sometimes returns to the room or seat on their own after confirming the source.
+1	When distracted by a sound, the child is sometimes able to remain seated if given individual verbal prompts.
+2	There are days when the child can focus on the task without being disturbed by surrounding sounds.
Post-intervention result: 0	

GAS: Goal Attainment Scaling.

**Table 5:** Comparison of Pre- and Post-Intervention Results by outcome measure (n = 10).

	Mean (Standard Deviation)				Difference CI 95%	Effect Size (r)	r CI 95%	*p*-Value
	Pre-Intervention		Post-Intervention		(Lower, Upper)		(Lower, Upper)	
GAS T-score	35.3	(0.5)	61.2	(7.7)	20.37, 31.43	0.889	0.269, 1.000	0.005 **
VABS-II								
Composite Adaptive Behavior Score	52.9	(9.8)	56.3	(9.8)	2.17, 4.63	0.893	0.274, 1.000	0.005 **
Domain standard score								
Communication	55.6	(13.8)	59.6	(15.4)	1.62, 6.38	0.779	0.179, 1.000	0.012 *
Subdomain v-scale score								
Receptive Language	6.1	(3.5)	8.4	(3.2)	1.08, 3.52	0.853	0.234, 1.000	0.007 **
Expressive Language	7.7	(3.0)	8.3	(3.1)	0.23, 0.97	0.775	0.155, 1.000	0.014 *
Reading and Writing	12.3	(3.4)	11.6	(3.4)	−1.29, −0.11	0.668	0.048, 1.000	0.035 *
Domain standard score								
Daily Living Skills	49.5	(13.7)	52.3	(15.3)	0.90, 4.70	0.754	0.134, 1.000	0.017 *
Subdomain v-scale score								
Personal	7.2	(3.2)	7.9	(3.6)	−0.31, 1.71	0.478	−0.142, 1.000	0.131
Domestic	8.1	(1.8)	8.6	(1.8)	−0.01, 1.01	0.598	−0.022, 1.000	0.059
Community	7.1	(2.5)	7.5	(3.1)	−0.10, 0.90	0.517	−0.103, 1.000	0.102
Domain standard score								
Socialization	40.6	(11.4)	45.9	(11.4)	3.42, 7.18	0.888	0.268, 1.000	0.005 **
Subdomain v-scale score								
Interpersonal Relationships	8.4	(3.3)	9.2	(2.3)	−0.14, 1.74	0.582	−0.038, 1.000	0.066
Play and Leisure	2.6	(2.3)	3.4	(2.6)	0.06, 1.54	0.652	0.032, 1.000	0.039
Coping Skills	7.3	(2.0)	8.6	(1.8)	0.62, 1.98	0.811	0.191, 1.000	0.010 *
Domain standard score								
Motor Skills	61.0	(16.5)	63.5	(16.7)	−1.29, 6.29	0.572	−0.048, 1.000	0.071
Subdomain v-scale score								
Gross Motor	10.1	(1.8)	10.6	(1.8)	-0.77, 1.77	0.200	−0.420, 0.820	0.527
Fine Motor	9.4	(2.2)	9.9	(2.1)	0.12, 0.88	0.707	0.087, 1.000	0.025
SSP Total Score	66.2	(17.1)	71.9	(16.4)	−2.51, 13.91	0.485	−0.135, 1.000	0.125
PSI-SF Total Score	49.8	(10.1)	50.2	(8.4)	−2.68, 3.48	0.288	−0.331, 0.908	0.362

* *p* < 0.05; ** *p* < 0.01. v-scale scores: mean = 15, SD = 3. Wilcoxon signed-rank test (effective sample size: 7–10). Effect size (r) calculated based on the total sample (n = 10). SSP: Short Sensory Profile; PSI-SF: Parenting Stress Index, Short Form; VABS-II: Vineland Adaptive Behavior Scales, Second Edition.

## Data Availability

The data presented in this study are available on request from the corresponding author due to privacy and ethical restrictions.

## Supplementary Materials

The following supporting information can be downloaded at: https://www.mdpi.com/article/10.3390/children13040569/s1, Table S1: Participant characteristics and details of concurrent services. Frequency is expressed as sessions per week and duration as minutes per session. Table S2: Individual pre-post changes in outcome measures.

## Abbreviations

The following abbreviations are used in this manuscript:

ASD	Autism Spectrum Disorder
OT	Occupational therapy
OTR	Occupational Therapist Registered
SIT	Sensory Integration Therapy
ID	Intellectual Disability
DSM-5	Diagnostic and Statistical Manual of Mental Disorders, Fifth Edition
CARS2	Childhood Autism Rating Scale Second Edition
CARS2-ST	CARS2-Standard Version
DQ	Developmental Quotient
KSPD	Kyoto Scale of Psychological Development
DA	Developmental Age
GAS	Goal Attainment Scaling
COPM	Canadian Occupational Performance Measure
VABS-II	Vineland Adaptive Behavior Scales, Second Edition
SSP	Short Sensory Profile
PSI-SF	Parenting STRESS index short form
PSI	Parenting STRESS index
