# Supplementary material for: Sensory Integration Therapy for Preschool Children with Autism Spectrum Disorder and Co-Occurring Intellectual Disability: An Exploratory Single-Group Pre–Post Study
Source: Children (Basel). 2026 Apr 20;13(4):569. doi: 10.3390/children13040569 (PMC13115176; doi:10.3390/children13040569)
Supplement: Supplementary file 1 [file children-13-00569-s001.zip › children-4217156-supplementary.pdf]

Supplementary Table S1. Participant characteristics and details of concurrent services. Frequency is expressed as sessions per week and duration as minutes per session.

Case	Age (months)	DQ	CARS	Nursery School/ Kindergarten	Ryoiku				
					Individual 1	Individual 2	Individual 3	Group 1	Group 2
1	71	67	40.5	5/w	1/w, 60 min			1/w, 90 min	
2	65	65	38	5/w	1/w, 60 min				
3	51	70	38.5	3/w	1/w, 60 min			2/w, 240 min	
4	58	69	43	5/w				1/w, 60 min	
5	47	61	39.5		2/w, 120 min			2/w, 270 min	
6	51	63	38.5					4/w, 300 min	
7	52	55	38					5/w, 240 min	
8	58	65	41	5/w					
9	49	64	39.5	3/w	3/w, 50 min	1/w, 70 min	1/w, 60 min	3/w, 120 min	
10	60	41	41	5/w				1/w, 120 min	1/w, 180 min

DQ: Developmental Quotient.

CARS: Childhood Autism Rating Scale; higher scores indicate greater autism severity.

Ryoiku: Structured developmental support services provided outside nursery/kindergarten.

Frequency and duration: Expressed as sessions per week (w) and minutes per session (min).

Supplementary Table S2. Individual pre–post changes in outcome measures.

Case	GAS T-score			VABS-2 Composite Adaptive Behavior Score			SSP Total Score			PSI-SF Total Score		
	pre	post	change	pre	post	change	pre	post	change	pre	post	change
1	35	57	22	42	48	6	102	87	-15	62	52	-10
2	35	72	37	52	55	3	72	69	-3	51	51	0
3	35	61	26	61	67	6	72	88	16	48	51	3
4	36	64	28	42	45	3	77	79	2	39	41	2
5	35	57	22	60	63	3	40	42	2	36	41	5
6	35	76	41	60	65	5	47	50	3	35	35	0
7	36	59	23	52	53	1	63	65	2	54	58	4
8	36	59	23	60	62	2	63	90	27	61	60	-1
9	35	57	22	64	66	2	69	82	13	59	57	-2
10	35	50	15	36	39	3	57	67	10	53	56	3

GAS (Goal Attainment Scaling) T-score: Standardized score with a mean of 50 and SD of 10; higher scores indicate greater goal attainment.

VABS-2 (Vineland Adaptive Behavior Scales, Second Edition): Composite Adaptive Behavior Score; higher scores indicate better adaptive functioning.
SSP (Short Sensory Profile): Total score; higher scores indicate greater sensory processing difficulties. Total scores were categorized as follows: 38–57 = typical performance, 58–70 = high, and 80–190 = very high.
PSI-SF (Parenting Stress Index–Short Form): Total score; higher scores indicate greater parenting stress.
